# Investigating the Relationship Between Persistent Reflux Flow on the First Postoperative Day and Recurrent Varicocele in Varicocelectomy Patients

**DOI:** 10.14740/jocmr1967w

**Published:** 2014-10-16

**Authors:** Ahmet Said Cil, Murat Bozkurt, Duygu Kara Bozkurt, Mustafa Gok

**Affiliations:** a Department of Radiology, Universal Hospital Groups, Malatya, Turkey; b Department of Obstetrics and Gynecology, Kafkas University School of Medicine, Kars, Turkey; c Department of Radiology, Kafkas University School of Medicine, Kars, Turkey

**Keywords:** Color Doppler sonography, Varicocele, Reflux

## Abstract

**Background:**

The aim of this study was to investigate the presence of persistent reflux flow on the first postoperative day using color Doppler sonography (CDS) in patients who had undergone sub-inguinal varicocelectomy, and to research the relationship between persistent reflux flow and recurrent varicocele.

**Methods:**

A total of 54 patients were included in the study. Ages of the patients were between 21 and 38 years (mean 27.3 ± 7.6). All patients were evaluated four times with CDS: preoperatively, first postoperative day, 3 months postoperative, and finally 6 months after the operation.

**Results:**

Preoperative venous diameters were measured between 3 and 5.5 mm; mean vein diameters were 3.8 ± 0.7 mm for the left side and 3.4 ± 0.4 mm for the right side. Mean duration of reflux was 3.5 ± 0.3 seconds on the left side and 2.9 ± 0.7 seconds on the right side. First postoperative day persistent Valsalva-induced reflux flow was seen in 10 patients (18%). Mean venous diameter was measured 1.8 ± 0.9 mm. Three months after the operation, Valsalva-induced reflux flow was seen in two patients (3%) in whom reflux was not seen on the first postoperative day. After 6 months, venous diameters larger than 2 mm at rest and the occurrence of reflux during the Valsalva maneuver were considered to be a recurrence. Six months after the operation, 12 patients had recurrent varicocele. Detecting persistent reflux with CDS on the first postoperative day was found to be 85% sensitive and 100% specific for showing recurrence.

**Conclusion:**

Valsalva-induced persistent reflux flow investigated with CDS on the first postoperative day can be used to show success of the surgery and is also an indicator of recurrence in varicocelectomy patients.

## Introduction

Varicocele is an abnormal dilation of the testicular veins in the pampiniform venous plexus and is the most common treatable cause of male infertility [1]. There are several surgical techniques defined for treatment. Depending on the surgical technique used, recurrence is seen in 1-45% of patients [2]. There is not an accepted imaging method for evaluation of surgical success and detection of recurrence in the early postoperative period. In this study, we aimed to determine the diagnostic value of investigating Valsalva-induced persistent reflux flow on the first postoperative day with color Doppler sonography (CDS) for evaluating surgical success and detection of recurrence in sub-inguinal varicocelectomy patients.

## Material and Methods

Between 2011 and 2013, a total of 54 patients aged 21 - 38 years (mean 27.3 ± 7.6) admitted to our urology and gynecology clinics with complaints of scrotal pain or infertility were included in the study. All patients had palpable grade III varicocele on physical examination according to the grading system described by Dubin and Amelar [3]. On CDS examination, all patients had veins larger than 3 mm in diameter at rest and more than 1 s reflux was present during the Valsalva maneuver (increase the intra-abdominal pressure with a closed epiglottis just after a deep inspiration). None of the patients had previously undergone varicocelectomy or urogenital surgery. Recurrent varicocele was defined as venous diameters larger than 3 mm at rest and occurrence of reflux longer than 1 s during the Valsalva maneuver 6 months after the operation. Our institutional review board approved the study. All patients were orally informed about the study. All patients underwent sub-inguinal varicocelectomy by the same surgeon without use of an optic magnifier. The surgeon was not informed about the study. Because its recurrence rates are higher than with other methods, only sub-inguinal varicocelectomy patients were included in the study. Subclinical varicocele patients were not included the study.

The same radiologist performed preoperative and postoperative CDS examinations using LOGIQ 9 (GE Healthcare Technologies, Ultrasound, Milwaukee, USA) and linear (12 - 14 MHz) transducer. In the supine position, testicular and epididymal size and specifications were recorded for all patients. While the patient was standing, diameters of venous structures were measured at the level of the distal end of the inguinal channel and superolateral aspects of the testicles before and after the Valsalva maneuver. Presence and duration of reflux were assessed in spectral analysis. CDS examinations were made for all patients before the operation, on the first postoperative day and 3 and 6 months after the operation. Findings were evaluated with a personal computer and SPSS software.

## Results

**Figure 1:**
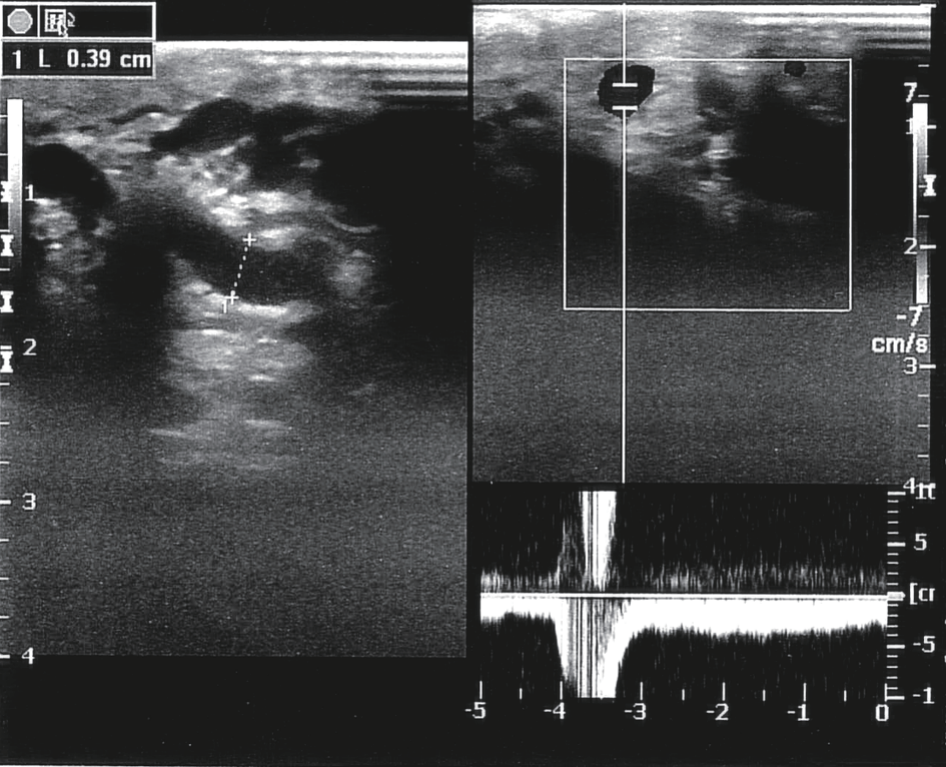
Preoperative CDS of a patient shows dilated testicular veins and Valsalva-induced reflux flow in duplex mode.

Preoperatively, 45 patients had left side and nine patients had bilateral grade III varicocele. Preoperative vein diameters were measured 3 - 5.5 mm, with mean vein diameters of 3.8 ± 0.7 mm for the left side and 3.4 ± 0.4 mm for the right side (Fig. 1). Mean duration of reflux on the left side was 3.5 ± 0.3 s and 2.9 ± 0.7 s on right side (Table 1).

**Table 1:** Preoperative CDS Findings

	Number of patients (n)
Vein diameters (mm)	
3.5 - 4	42
4 - 4.5	7
> 4.6	5
Localization	
Left	45
Bilateral	9

**Figure 2:**
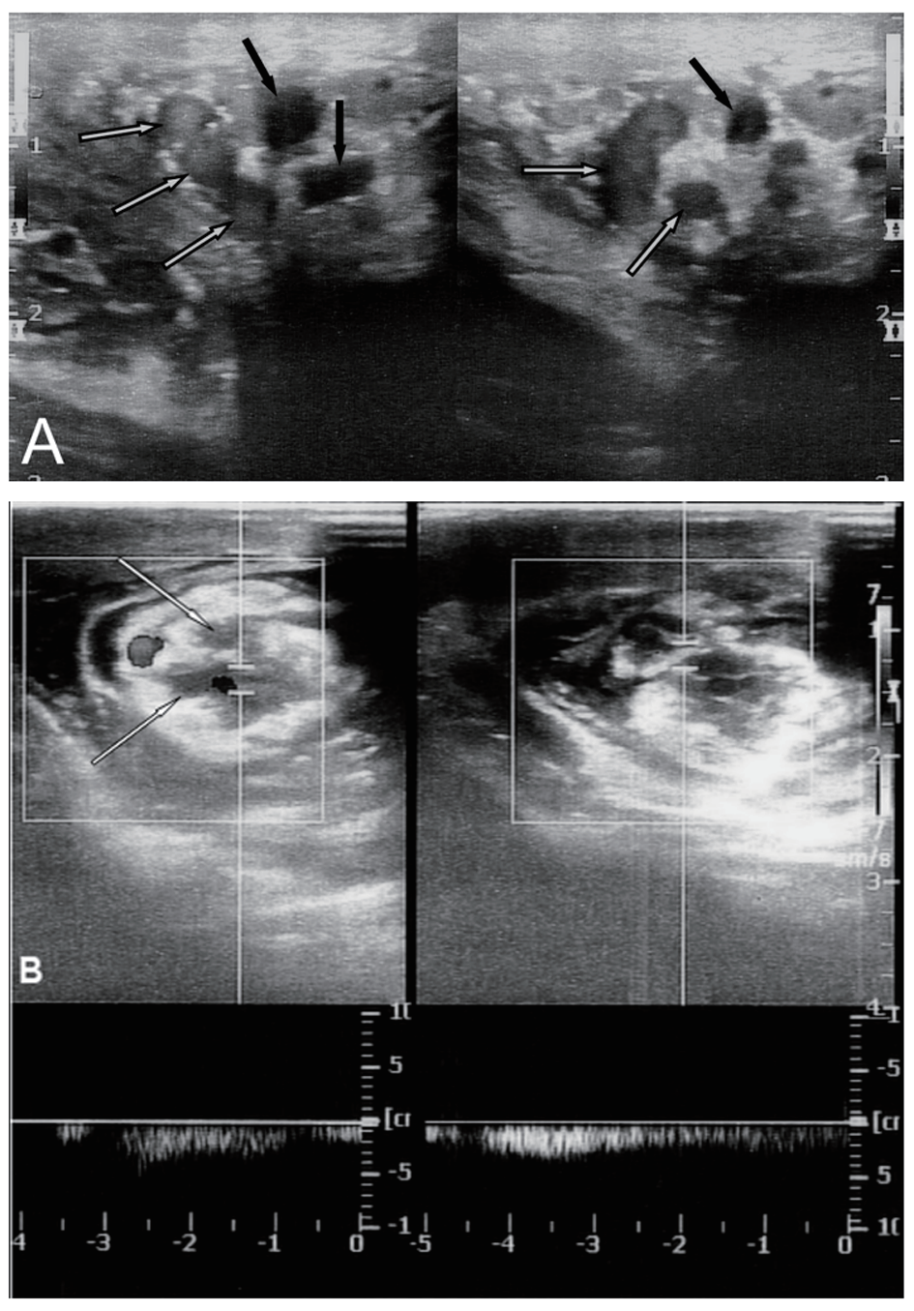
(A) Postoperatively first day US of a patient shows thrombosed veins (white arrows) and open veins (black arrows). Edematous changes were seen around the venous structures. (B) CDS shows Valsalva-induced persistent reflux in duplex mode. Note the edematous changes around the venous structures.

**Figure 3:**
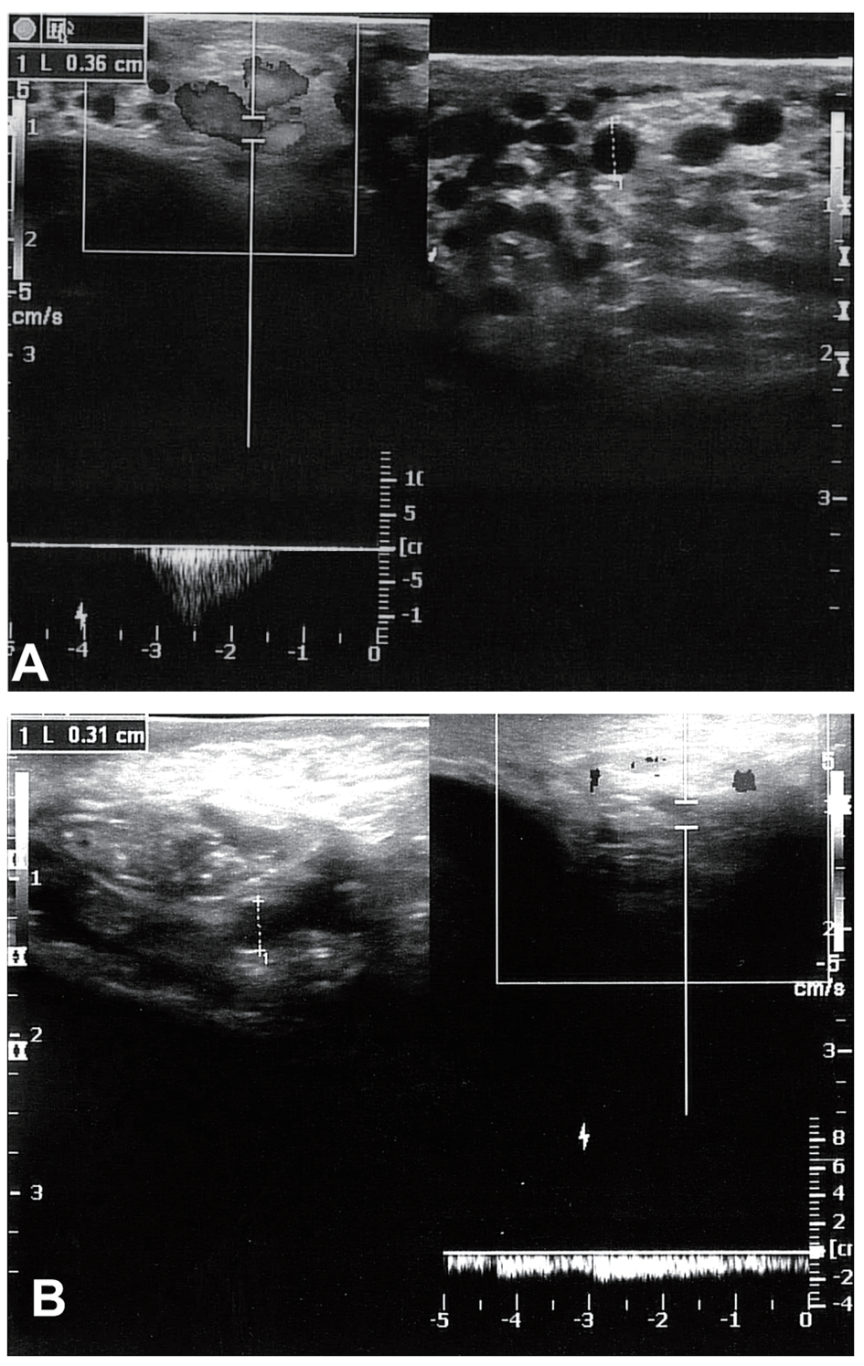
CDS images of two different patients 6 months after surgery show Valsalva-induced reflux flow and recurrent varicocele.

On the first postoperative day, CDS examinations, edematous changes in soft tissues around the spermatic cord, and presence of thrombus in some testicular veins were seen in all patients. With the patient standing, persistent reflux was seen in 10 patients (18%) in the non- thrombosed open veins with the Valsalva maneuver (Fig. 2). The diameters of veins in which reflux flow was observed were measured 1.6 - 2.5 mm (mean 1.8 ± 0.5 mm). Three months after surgery, all 10 of these patients had reflux longer than 2 s in pampiniform plexus veins and vein diameters measuring between 2.8 and 3.2 mm (mean 2.8 ± 0.5 mm). Valsalva-induced reflux flow was seen at 3 months after the operation in two patients (3%) who had had no reflux on the first postoperative day on CDS examination. Vein diameters were measured 2.4 - 2.6 mm (mean 2.5 ± 0.4 mm) in these patients. According to this criterion, 12 patients had recurrent varicocele (Fig. 3). Compared with the preoperative period, venous diameters and severity of reflux were decreased in all 12 of these patients (Table 2).

**Table 2:** Postoperative CDS Findings of Patients Who Had Persistent Reflux and Recurrent Varicocele

Reflux	Number of patients (n)	Age (years)	Vein diameter (mm)			
			Pre-op	Post-op		
				First day	Third month	Sixth month
First day	1	26	3.6	1.8	2.6	3.1
	2	22	4.2	2	2.8	3.3
	3	28	3.5	1.5	2.3	3.0
	4	30	4.2	1.6	2.3	3.2
	5	24	4.2	2.1	2.8	3.6
	6	26	5.3	1.5	3.2	4.0
	7	34	3.3	1.7	2.6	3.0
	8	26	3.5	1.9	2.6	3.2
	9	26	3.8	1.8	2.8	3.5
	10	29	3.7	2.3	2.9	3.2
Third month	11	25	4.6	2.1	2.4	3.1
	12	19	4.5	1.9	2.6	3.2

Persistent reflux and recurrence were not seen in the other 42 patients. Compared with the preoperative period, vein diameters and severity of reflux were decreased in all 42 of these patients at the sixth month after surgery as well. The recurrence rate was found to be 22% in patients who underwent sub-inguinal varicocelectomy. There was statistical significance (P < 0.01) between first postoperative day reflux and recurrence. Reflux on the first postoperative day was found to be 85% sensitive and 100% specific for determining recurrence.

## Discussion

In this present study, we have detected persistent reflux with CDS on the first postoperative day to be 85% sensitive and 100% specific for showing recurrence. We chose to look at only the macroscopic sub-inguinal varicocelectomy method due to its higher recurrence rates compared to other methods.

Surgery is the most commonly used treatment for varicocele patients. Although several surgical methods have been defined for varicocele treatment, in recent years, open inguinal or sub-inguinal microsurgery (varicocelectomy assisted by an optic magnifier) has been accepted to be the best choice due to its low complication and recurrence rates [4, 5].

Recurrence is one of the most unwanted results of varicocelectomy operations. In different reports, recurrence rates have varied between 1% and 45% [1]. The most frequent causes of recurrence are shunt development through the external spermatic vein, or unseen and non-ligated spermatic vein branches [5, 6].

Moon et al investigated the causes of varicocele recurrence and performed angiography on macroscopic sub-inguinal varicocelectomy patients. They found the mean varicocele recurrence time was 5.3 months (range: 0.75 - 13 months). They identified patent internal spermatic veins in 11 patients and treated using embolization [7].

Niedzielski et al used intraoperative venography for detecting non-ligated vessels in the varicocele repair operation. They detected non-ligated vessels in 21 patients (12%) via venography, which were revised and repaired during the same procedure. They suggest that using intraoperative venography decreases the recurrence rate [8].

According to these studies, inadequate surgical procedure had a great role in recurrence development, and some of the spermatic vein branches may not be visible during the operation. In the present study using CDS, we observed persistent reflux flow in 10 patients on the first postoperative day, and in two patients 3 months after surgery. Although CDS does not show the communication and extensions, we surmised that these persistent reflux flow-containing veins on the first postoperative day are patent spermatic vein branches.

The aim of varicocelectomy is the ligation of all internal and external spermatic vein branches while separating vas deferens, lymphatic vessels and artery of the spermatic cord. Also, vessels of the vas deferens must be preserved and testicular venous drainage must remain intact [4]. Using an optic magnifier is the most practical method for enabling the separation of these structures and increasing operative success [4-6].

CDS is the most common and inexpensive method used worldwide for diagnosing varicocele and can be used on the first postoperative day for determining operative sufficiency. CDS may be a part of early postoperative follow-up as well as preoperative examination, especially for macroscopic surgeries. Although investigating persistent Valsalva-induced reflux flow on the first postoperative day with CDS may be used for determining patent spermatic veins, revision or correction is not possible with this method. Revision and correction are enabled by intraoperative venography. However, it is not practical for common use because it is expensive, requires specialized equipment, and has high morbidity. There is still not an accepted non-invasive and commonly useable imaging technique for determining patent spermatic veins intraoperatively.

In conclusion, investigating persistent Valsalva-induced reflux flow on the first postoperative day with CDS may show operative sufficiency, and the presence of persistent reflux on the first postoperative day may be used as an indicator of recurrence. However, this method does not help to decrease recurrence rates.

## References

[1] Cayan S, Kadioglu A (2005). Current approaches in the diagnosis and treatment of varicocele. Turk Uroloji Dergisi.

[2] Tefekli A, Cayan S, Uluocak N, Poyanli A, Alp T, Kadioglu A (2001). Is selective internal spermatic venography necessary in detecting recurrent varicocele after surgical repair?. Eur Urol.

[3] Dubin L, Amelar RD (1970). Varicocele size and results of varicocelectomy in selected subfertile men with varicocele. Fertil Steril.

[4] Abdel-Maguid AF, Othman I (2010). Microsurgical and nonmagnified subinguinal varicocelectomy for infertile men: a comparative study. Fertil Steril.

[5] Goldstein M, Gilbert BR, Dicker AP, Dwosh J, Gnecco C (1992). Microsurgical inguinal varicocelectomy with delivery of the testis: an artery and lymphatic sparing technique. J Urol.

[6] Cayan S, Kadioglu TC, Tefekli A, Kadioglu A, Tellaloglu S (2000). Comparison of results and complications of high ligation surgery and microsurgical high inguinal varicocelectomy in the treatment of varicocele. Urology.

[7] Moon KH, Cho SJ, Kim KS, Park S (2012). Recurrent varicoceles: causes and treatment using angiography and magnification assisted subinguinal varicocelectomy. Yonsei Med J.

[8] Niedzielski J, Paduch DA (2001). Recurrence of varicocele after high retroperitoneal repair: implications of intraoperative venography. J Urol.

